# Editorial: Synthetic biology approaches for stress adaptation and improved metabolism in microorganisms

**DOI:** 10.3389/fbioe.2022.1013073

**Published:** 2022-09-08

**Authors:** Chong Li, Xiaofeng Yang

**Affiliations:** ^1^ Shenzhen Branch, Guangdong Laboratory for Lingnan Modern Agriculture, Shenzhen Key Laboratory of Agricultural Synthetic Biology, Genome Analysis Laboratory of the Ministry of Agriculture and Rural Affairs, Agricultural Genomics Institute at Shenzhen, Chinese Academy of Agricultural Sciences, Shenzhen, China; ^2^ Kunpeng Institute of Modern Agriculture at Foshan, Chinese Academy of Agricultural Sciences, Foshan, China; ^3^ School of Biology and Biological Engineering, South China University of Technology, Guangzhou, China

**Keywords:** microbial adaptation, stress tolerance, microbial inhibition, metabolic pathway, adaptive evolution

Environmental deterioration and energy shortage have promoted the emergence of biorefinery. However, in the fermentation process of biorefinery, microbes are usually subjected to adverse environmental stresses, which negatively affect cell growth and productivity, ultimately limiting the development of green biomanufacturing. Therefore, exploration of approaches for stress adaptation and improved metabolism in microorganisms is of great significance. The current research topic aims to reveal the damaging effects of environmental stresses on microbial strains, and more importantly, introduce advanced strategies, including microbial adaptive evolution, fermentation strategy innovation, reprogramming of genetic circuits to improve the tolerance of microorganisms to adverse conditions. Meanwhile, with the development of emerging techniques, multi-omics such as global transcriptome, proteome and metabolome have been used for elucidation of the mechanisms of stress tolerance in microbes. It is therefore vitally important for the rational design and construction of the strains with a desirable phenotype of stress tolerance.

For directly improving the tolerance of microorganisms to adverse environmental stresses, adaptive evolution is a useful strategy to evolve microorganisms under specific environmental conditions, including high substrate/product concentration, high temperature, and low/high pH (Li et al., 2018, 2019; Sandberg et al., 2019). In the study of Zhou et al., a strategy named microbial microdroplet culture (MMC) was applied for the adaptive evolution of *G. oxydans* to improve its tolerance to high substrate concentration and temperature. Compared with the traditional cultivation methods, MMC has high throughput, less reagent and labor cost, and superior cultivation properties in mixing and parallelization (Jian et al., 2020). As a result, the strain MMC10 that can grow in a high concentration of 300 g/L of D-sorbitol medium at 40°C was achieved.

The development of multi-omics techniques in recent years have significantly facilitated the studies in engineering of microbial stress resistance. In, Zhao et al. from Shanghai Jiao Tong University identified novel zinc-responsive proteins that play important roles in acetic acid tolerance of *S. cerevisiae* by comparative proteomics. The overexpression of two identified genes including *KIC1* and *CDC42* endowed *S. cerevisiae* with faster growth and ethanol fermentation under the stress of acetic acid and mixed inhibitors in substrate of cellulosic hydrolysate. The accumulation of end-products always leads to osmotic stress and hinders further increase of the product. In the manuscript of, Xu et al. from Tianjin Institute of Industrial Biotechnology (CAS) conducted a comparative transcriptomic analysis to determine the gene expression profiles of *C. glutamicum* under high-lysine stress conditions. This study found several possible metabolic pathways that might offer favorable benefits for high-lysine adaptation. In the meanwhile, they identified a GrpE chaperone that confers high-lysine stress tolerance in *C. glutamicum* and demonstrated the importance of DNA repair component and energy transducing NADH dehydrogenase for protecting cells against the osmotic stress. Taken together, the multi-omics analysis will guide the rational design and construction of the strains against adverse condition stress.

Apart from the adaptation of adverse conditions, microorganisms evolve strategies to tolerant against the stresses. In the study of, You et al., identified a single-point mutation in ClpX could boost the motility of *E. coli* via mediating the accumulation of the master regulator FlhDC, which might be an optional strategy that strains could take.

In wine production, the metabolism of organic acid by microorganism plays a significant role in cell growth, metabolism, and wine quality improvement. During this process, the uptake of extracellular organic acids by the transporters is the first rate-limiting step. In the study of, Liu et al. verified the function of a predicted citrate transporter gene JKL54_04345 (*citP*) in *L. plantarum* for the first time. In another food industry, amino acid production, a study of was reported by Zhen et al. from South China University of Technology. This study identified a nitrogen consumption regulon AmtR in the industrial producer of L-glutamate, *C. glutamicum*. The AmtR regulator could enable the strain to grow in medium with creatine, a nitrogen source that could not be consumed originally, as a sole nitrogen source, which provides a strategy for strains to survive in a medium without suitable nutrition. The enhancement of substrate transportation or consumption can enhance the interaction between strains and the surroundings and thus alleviate the environmental stress.

As an ideal protein source that is consumed, the unicellular green alga *Chlorella* provides high protein content with a balanced amino acid composition, but suffers from low production rate and high cost owing to its low salt tolerance (Liu and Hu, 2013). In order to overcome the problem, Jin et al. reported the ultrahigh-density fermentation of *Chlorella sorokiniana* with an innovation in nitrogen supply and strict control in fermentation parameters. This may enlighten the heterotrophic cultivation of other freshwater microalgae with similar problems.

There are still technical challenges have proven difficult to overcome, in part, because of inherent limitations in host organisms. Efforts have been made, but success is often limited because these microbes are frequently polygenic, requiring the manipulation of multiple genes to bring about substantial changes. Moreover, the detailed molecular mechanisms underpinning phenotype are not always fully understood. Nevertheless, the development of the multi-omics techniques may contribute to the rational construction of the strains against adverse condition stress from a global pattern.
